# Swept-Source-Based Chromatic Confocal Microscopy

**DOI:** 10.3390/s20247347

**Published:** 2020-12-21

**Authors:** Dawoon Jeong, Se Jin Park, Hansol Jang, Hyunjoo Kim, Jaesun Kim, Chang-Seok Kim

**Affiliations:** 1 Department of Cogno-Mechatronics Engineering, Pusan National University, Busan 46241, Korea; 2atdawn@pusan.ac.kr (D.J.); eldorajin@pusan.ac.kr (S.J.P.); hsjang888@pusan.ac.kr (H.J.); 2 Taihan Fiber Optics Co., Ltd., Ansan-si 15601, Gyeonggi-do, Korea; hyunjoo.kim@tfo.co.kr (H.K.); jaesun.kim@tfo.co.kr (J.K.)

**Keywords:** chromatic confocal microscopy, chromatic aberration, swept-source, wavelength-swept laser, high-speed, 3D surface profile

## Abstract

Chromatic confocal microscopy (CCM) has been intensively developed because it can exhibit effective focal position scanning based on the axial chromatic aberration of broadband light reflected from a target. To improve the imaging speed of three-dimensional (3D) surface profiling, we have proposed the novel concept of swept-source-based CCM (SS-CCM) and investigated the usefulness of the corresponding imaging system. Compared to conventional CCM based on a broadband light source and a spectrometer, a swept-source in the proposed SS-CCM generates light with a narrower linewidth for higher intensity, and a single photodetector employed in the system exhibits a fast and sensitive response by immediately obtaining spectrally encoded depth from a chromatic dispersive lens array. Results of the experiments conducted to test the proposed SS-CCM system indicate that the system exhibits an axial chromatic focal distance range of approximately 360 μm for the 770–820 nm swept wavelength range. Moreover, high-speed surface profiling images of a cover glass and coin were successfully obtained with a short measurement time of 5 ms at a single position.

## 1. Introduction

Three-dimensional (3D) surface imaging is becoming increasingly important in various fields, such as the surface defect testing of tiny electronic components or measuring the 3D structure of living cells; consequently, a fast and accurate surface profiling technique is in high demand [1,2,3,4]. Among the several types of methods available for 3D surface profiling, optical technology, such as optical coherence tomography (OCT) and confocal microscopy (CM), has been noticed for its high throughput without any direct contact or damage to the target sample [5,6,7]. Compared to interferometric OCT imaging with a low numerical aperture (NA) lens, CM can provide a higher axial and lateral resolution if a high-NA lens and a limited depth of focus from a pinhole are used [8]. Despite these advantages, CM has suffered from imaging speed limitations due to its mechanical scanning process for the lateral and axial directions, and thus, there have been many attempts to improve its scanning speed.

Chromatic confocal microscopy (CCM) is one of the recently proposed methods for solving the problem of slow scanning speed; particularly, this method employs an optical technique to remove the mechanical axial scanning process [9]. The CCM uses chromatic aberration of the lens to generate light dispersion depending on wavelength, causing each wavelength to correspond to a different focal distance. Consequently, the focal distance of the lens is spectrally encoded, and the relative depth of the object can be obtained by decoding the wavelength information from the spectrum of the reflected light [10,11]. Over the years, the speed of imaging systems was improved by replacing the mechanical axial scanning process with chromatic aberration-based techniques. However, the problem of a low imaging speed still remains [12]. The conventional CCM, based on a broadband light source, has a low signal intensity. This is because the intensity of the light reflected from the target reduces when this total intensity is divided among each wavelength segment. In addition, the sensitivity of the spectrometer is insufficient with respect to the low-intensity of light in each wavelength segment. Thus, owing to a low signal-to-noise ratio (SNR), it is difficult to perform a single-shot detection or measurement of translucent samples, such as glass, with CCM [13]. As a result, signal integration was inevitably introduced to supplement the low SNR, which again led to the degradation of the measurement speed. A few methods have been suggested to replace the spectrometer of CCM, but it was not easy to obtain either high-speed or high sensitivity together by avoiding complicated setup [1,12,14,15]. Thus, a novel method for enhancing the measurement speed is required while maintaining sufficient sensitivity.

In OCT, under the category of spectral-domain OCT (SD-OCT), the combination of a broadband light source and a spectrometer has been used for obtaining the spectral information of the optical interference signal. Recently, a newer generation of swept-source OCT (SS-OCT) has been introduced because of its deeper penetration ability due to its higher sensitivity and faster acquisition time achieved by using only a photodetector (PD) [16]. In SS-OCT, the light source is already divided into a spectrum using a wavelength-swept laser, thus removing the necessity of a spectrometer. This simplified mechanism and the improved performance of the swept-source (SS) has already contributed to the rapid evolution of the OCT imaging system and other optical microscopy instruments. However, to the best of our knowledge, such an SS has not been applied to the development of CCM to date.

Thus, in this paper, we have proposed a novel configuration of swept-source-based chromatic confocal microscopy (SS-CCM) and demonstrated its excellent sensitivity and fast measurement speed simultaneously [17]. While most existing CCM systems use a white light source having a polychromatic broadband spectrum, the proposed SS-CCM employs a wavelength-swept laser as the light source. This SS generates light in a certain range of wavelengths rapidly, continuously, and repeatedly, resulting in an equivalent wavelength range similar to that of a broadband source. The generated light undergoes chromatic dispersion due to the chromatic probe and strikes the object to be imaged. The proposed SS-CCM system does not require a spectrometer to detect the reflected light because the underlying process is not wavelength-related but is time-related. Particularly, a PD can be employed for obtaining the required spectral information of the reflected light along the time scale repeatedly. Since the combination of an SS and a PD results in a sufficiently high SNR and detection sensitivity, it was theoretically and experimentally demonstrated in this work that the proposed SS-CCM can be successfully employed as a new high-speed 3D surface profiling tool.

## 2. Theory

**Figure 1:**
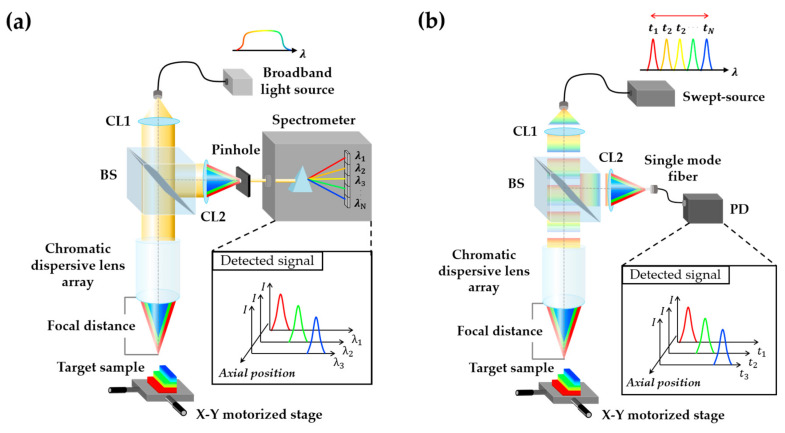
Schematic diagrams and data processing procedure of (**a**) the conventional chromatic confocal microscopy (CCM) and (**b**) the proposed swept-source-based CCM (SS-CCM). CL, BS, and PD are the acronyms for collimation lens, beam splitter, and photodetector, respectively.

In conventional CCM, the axial position of a reflecting surface is obtained from the relative spectral intensity distribution of the broadband light reflected off a series of lenses having a high degree of chromatic aberration. As shown in Figure 1a, once the broadband light enters inside the chromatic dispersion probe, different wavelengths are focused on the different axial positions due to chromatic aberration and the reflected light is filtered by a pinhole. When a single wavelength of the broadband light is in focus, whereas the other wavelength components are out of focus, it is possible to induce an intensity distribution along the wavelength using a spectrometer. The focused wavelength component passes predominantly through the pinhole and corresponds to an axial reflecting position of the target surface. Ideally, the chromatic probe is designed to have a focal distance that varies linearly with wavelength and to collect the spectral intensity distribution data using the spectrometer. Depending on the wavelength of light, the focal distance,$\;f\left(\lambda \right)$, can be converted into the relative axial reflecting position using the following equation [11,18,19].

(1) $$f\left(\lambda \right)=f_{0}+S\Delta \lambda$$

where $f_{0}$ is the minimum focal distance matching the initial wavelength of the broadband light source, Δλ is the change in the wavelength, and S represents the chromatic slope, which corresponds to the change in the focal distance for a change in the wavelength. Following this relationship, the relative depth of the reflection surface can be acquired with only one-point measurement, and thus, a 3D image of an object can be constructed by combining the information at multiple depths obtained from scanning in the two lateral directions.

In the SS-CCM shown in Figure 1b, a chromatic probe identical to that used in a conventional CCM can be used. However, instead of the light generated from the broadband source, the light generated from a wavelength-swept laser within the swept-source is inputted into this probe. The light reflected from the target is collimated via a single-mode fiber, which plays a role similar to that of a pinhole in the conventional CCM, and the optical signal is detected by a PD instead of the spectrometer that is used in the conventional CCM. The data acquired using the PD is expressed using the intensity distribution as a function of time. Thus, a new equation is required for determining the relationship between the periodic time of the swept-source time and focal distance. The central wavelength from the swept-source can be represented by Equation (2):

(2) $$\lambda \left(t\right)=\lambda_{sweep}+\Delta \lambda =\lambda_{sweep}+R_{sweep}\Delta t$$

(3) $$f\left(t\right)=f_{0}+SR_{sweep}\Delta t$$

where $R_{sweep}$ and $\lambda_{sweep}$ correspond to the sweep speed and the starting sweep wavelength of the swept-source, and Δt is a time increment. Equation (2) can be applied to alternate the change in the wavelength in Equation (1) successfully. Assuming that the initial wavelength of the broadband light source and starting sweep wavelength of the swept-source are identical, the focal distance can be expressed as a function of the periodic time of the swept-source, as given in Equation (3). Thus, in the SS-CCM system, it is clearly allowed to convert the varying intensity over time into the relative depth of the reflection surface. Since the swept-source-based axial scanning process in combination with PD can eliminate the critical defects of conventional CCM, such as limited sensitivity and speed due to the spectrometer, it achieves a higher sensitivity and faster detection owing to the replacement of the spectrometer with a PD.

## 3. Experimental Setup

Referring to the schematic diagram of the proposed SS-CCM system shown in Figure 1b, a commercial wavelength-swept laser (BS-785-1-HP, Superlum, Ireland) was used as the swept-source with a central wavelength of 795 nm, a sweeping range of 50 nm, an average output power of 4.2 mW, and a sweep repetition frequency of 200 Hz, which is triggered by an electric signal from the swept-source. A collimation lens was used to increase the size of the light source, and the wavelength-swept light entered a 50:50 beam splitter. After passing through the beam splitter, the light was focused onto each axial position corresponding to the focal distance of each wavelength through the chromatic dispersive lens array. The light was reflected from the target sample onto the *X*-*Y* motorized stage and once again traveled through the chromatic probe in the reverse direction to reach the beam splitter. The other collimation lens was used for concentrating the focused light into the single-mode fiber, which delivered only the allowed light to the PD and blocked off the defocused light.

**Figure 2:**
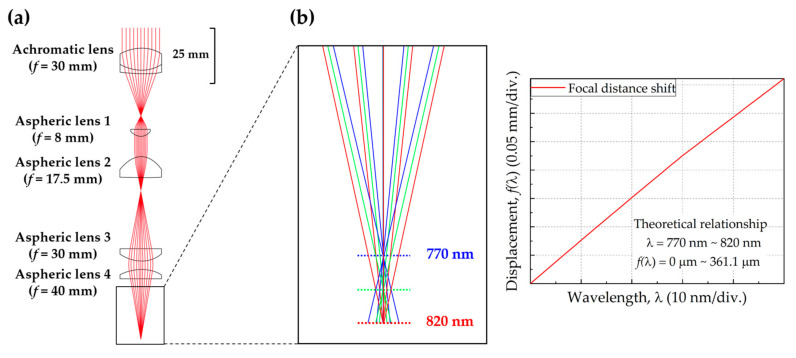
(**a**) Optical design of the chromatic dispersive lens array. (**b**) The relationship between the displacement and the wavelength of light corresponding to the chromatic dispersion simulation of the designed probe obtained using CODE V.

The chromatic dispersive lens array was designed using a few commercial lenses by employing the optical system simulation package CODE V (Optical Research Associates, Pasadena, CA) [20]. Since it is preferred to have a chromatic probe exhibiting large chromatic aberration as well as a small spherical aberration, acrylic-based aspherical lenses were selected. It is known that acrylic-based materials exhibit substantial chromatic aberration due to their low Abbe number and, at the same time, aspherical lenses reduce spherical aberration. To maximize the chromatic aberration in the wavelength range corresponding to that of the swept-source, between 770 nm and 820 nm, aspheric lenses with focal lengths of 8, 17.5, 30, and 40 mm were packed in regular order, as shown in Figure 2b. Depending on the value of Δλ, the change in the focal distance was simulated iteratively. The simulation result of the optimal condition is shown in the right of Figure 2b, which demonstrates that the change in the focal distance, 361.1 μm, corresponds to a change in the wavelength from 770 nm to 820 nm. This relation can be expressed with the second–order equation of $f\left(\lambda \right)=-14.0830+28,670.9\lambda -13483900\lambda^{2}\;\left(R^{2}=0.9999\right)$ or the first-order equation of $f\left(\lambda \right)=-5.56377+7231.62\lambda \;\left(R^{2}=0.9994\right)$ [21]. Since the difference between both focal distance equations is not so serious, it can be accepted that the linearity of the first-order equation can be tolerated as a function of the desired measurement precision.

## 4. Comparison between the Conventional CCM and SS-CCM

An experimental comparison between the conventional CCM and the proposed SS-CCM system was performed to evaluate the results objectively. The conventional CCM was easily built by replacing the swept-source laser with a broadband source (SOA-332, Superlum, Ireland) and the PD with a spectrometer (USB 2000+, OceanOptics, USA) from the established SS-CCM system. Since chromatic aberration is a function of the central wavelength and chromatic dispersion, the central wavelength of the broadband source was set at nearly 795 nm, equal to that of the swept-source. The bandwidth and average power of the broadband source output were 37 nm and 1.6 mW, respectively.

**Figure 3:**
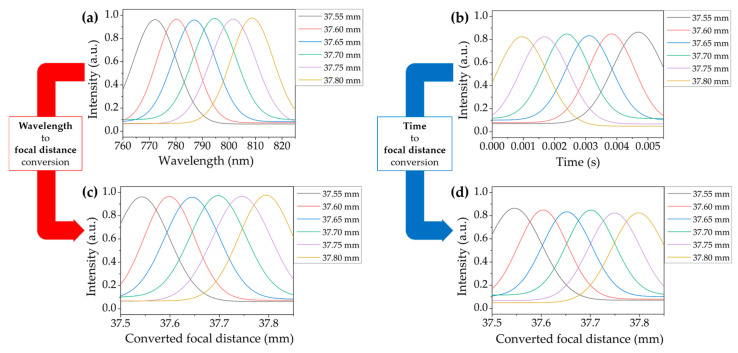
Measured intensity distribution (**a**) as a function of the wavelength obtained using the conventional CCM and (**b**) as a function of time obtained using the SS-CCM system. The converted information of the focal distance shift is shown in (**c**) for the conventional CCM and (**d**) for the SS-CCM system.

Figure 3 shows the experimental results of the focal distance shift obtained from the conventional CCM and the proposed SS-CCM system. Figure 3a,b correspond to the measured intensity distribution acquired by the conventional CCM and SS-CCM, respectively, by moving the mirror on the stage along the axial direction by 50 μm to 6 different positions from 37.55 mm to 37.80 mm. As the light from each light source underwent the intended chromatic aberration, it reached the target surface located at different axial positions and the reflected light bearing the spectrally encoded depth was repeatedly measured using either a spectrometer or a PD, respectively. The detected signals were normalized and subjected to Gaussian fitting. The results thus obtained are presented in the form of the intensity as a function of wavelength for the conventional CCM and intensity as a function of time for the SS-CCM in Figure 3a,b and the corresponding signals for the same depth were plotted using the same color.

Figure 3c,d shows the converted plots for each intensity as a function of the axial position, which was transformed using the focal distance equation of each system. For the conventional CCM, the focal distance shift was obtained by searching the peak intensity wavelength for the Gaussian signal from each axial position. A linear fit ($R^{2}=0.999)$ to the focal distance shift yielded the equation $f\left(\lambda \right)=6869.34\lambda +32.2392$, where 37.55 mm represents the shortest focal distance for the wavelength of λ≈0.000773 mm. Since the fitted equation satisfactorily follows the theoretical relationship between the wavelength and focal distance expressed by Equation (1), the peak intensity wavelength (in mm) detected by the spectrometer can be converted into a relative axial position (in mm) using the focal distance equation. An experiment was also performed to investigate the specifications of the SS-CCM. The reflected intensity detected by the PD as a function of time was converted to the relative axial position (in mm) using the experimentally obtained focal distance equation, $f\left(t\right)=37.8629-67.6987t$ ($R^{2}=0.999)$.

The accuracy of the axial optical scanning process in SS-CCM can be verified by comparing it with the corresponding results obtained using the conventional CCM shown in Figure 3c,d. The measured depth of the target surface can be obtained by extracting the central peak position from each plotted Gaussian function. The peak of each graph was converted to an axial position using the focal distance equations. Thus, it can be inferred that SS-CCM has a reliable accuracy, which is similar to that of the existing CCM technology, and it also stably covers an axial scanning range of approximately 250 μm. The axial resolution could be approximated with full-width at half maximum (FWHM) values, and the measured average FWHM values for each of the conventional CCM and SS-CCM were 139.308 μm and 131.452 μm, respectively. Compared to other reported CCM systems or commercial systems of which the axial resolution reaches about tens of nanometers, it shows that our system implemented a relatively lower level precision [15,18,22,23]. We suspect that axial resolution is mainly affected by the objective lens in our chromatic dispersive lens array. In particular, an aspheric lens 4 was not optimally designed yet to induce a high NA sufficiently. However, it is also noticed that the FWHM values of both conventional CCM and SS-CCM were not so significantly different because the same chromatic dispersive lens array was used in two systems. This supports that the proposed concept of replaced swept-source does not affect the maximum measurement precision of CCM in axial resolution.

Next, the varied accuracy, according to the minimally required acquisition time for single axial scanning, was explored in order to compare the performance of the two systems in depth. The acquisition time of each system was varied from 5 ms to 500 ms. A mirror sample was prepared as a target surface for the experiment. Under the sufficient intensity of light and high-reflectivity, it is not easy to explore the limit of imaging performance. To presume the situation with lower exposure intensity, a neutral density filter (NDL-25S-4, Thorlabs, Newton, NJ, US) of 0.8 optical density was mounted in front of the target to reduce the amount of receiving light. The distance of the target from the chromatic dispersive lens array was fixed to the same position during repeated measurements for different acquisition time durations. Similar to the previous experiment, the measured peak value along the wavelength and time was converted into the relative axial position using the focal distance relations given by the first experiment. The measured axial position of the target surface as a function of the acquisition time was traced by extracting the central peak value of the original signal.

**Figure 4:**
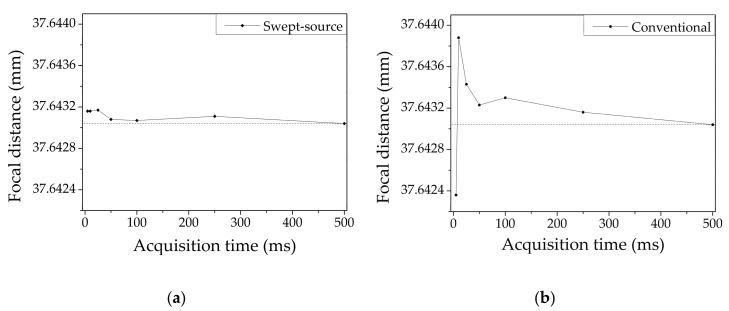
Relative distribution of the measured axial positions of the target surface as a function of the acquisition time for the (**a**) SS-CCM and (**b**) the conventional CCM.

To explore the performance differences in earnest for the same acquisition time conditions, the integration time of the spectrometer was tuned from 5 ms and 500 ms in the conventional CCM system, and the repetition speed of the swept-source was adjusted from 200 Hz to 2 Hz in the SS-CCM system. The measured axial positions of the target surface as a function of the acquisition time for the two systems are plotted in Figure 4 based on the axial position detected with the longest acquisition time for each system and assuming an acquisition time of 500 ms to be ideally sufficient for both the systems. A comparison within Figure 4a,b indicates that the SS-CCM system exhibits a stable distribution regardless of the acquisition time down to 5 ms, which corresponds to a single detection at a sweeping speed of 200 Hz. Since the maximum sweeping speed of the wavelength-swept laser is 200 Hz, 5 ms is the minimum acquisition time available and is the fastest condition of the designed system.

In contrast, in the case of the conventional CCM, the measurement results exhibit a larger variation and instability as compared to SS-CCM for each acquisition time condition. For acquisition times lower than 50 ms, severely inaccurate results are obtained owing to the limited number of photons and reduced signal sensitivity. In CCM technology, it is well known that the measurement is affected significantly by the reflectance of the samples and the sensitivity of the detecting device. In this regard, the problem of a low-reflectance sample requiring slow speed to gather a sufficient number of photons arises. However, our SS-CCM system exhibits remarkable performance with high stability as well as rapid speed even with a low-reflectance sample. This proves the potential of the proposed SS-CCM system for accurate high-speed imaging of various types of samples.

## 5. Imaging Results and Discussion

The imaging results were obtained from the 3D measurements performed using the proposed SS-CCM in combination with a commercial chromatic probe (IFS2405, Micron-epsilon, Germany). By replacing the original lab-built chromatic dispersion probe, the free space alignment was also modified to the all-fiber configuration by substituting the beam splitter and the collimation lenses with an optical circulator and single-mode fibers. First, a cover glass (HSU-010130, Superior, Germany) stacked on a glass slide was used as the target sample, providing a depth difference of 170 μm, which corresponds to its thickness, with 4% of reflectance from the surface. The lateral range of 2.6 mm, including the depth step between the cover glass and the glass slide, was scanned along 520 pixels for 5 ms for the single axial scanning of each pixel by moving the motorized stage and using a time interval of 5 ms between each pixel measurement. Lateral movement with an interval of 5 μm provided an excellent lateral resolution of a few microns. For the 2.6 mm lateral scanning range, the image was constructed by mapping the axial positions of the detected signals obtained after every 5 ms per pixel for a total imaging time of 5.195 s.

**Figure 5:**
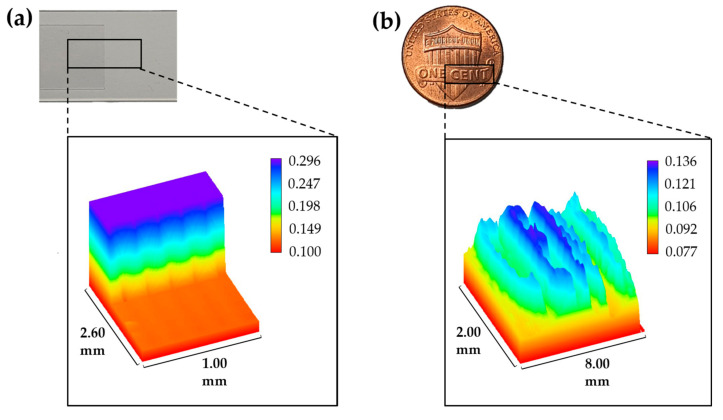
Three-dimensional (3D) surface profile images of (**a**) a cover glass stacked on a glass slide and (**b**) a “US cent” coin measured using the SS-CCM system.

Stable lateral bidirectional scans were also performed with a wider interval of 100 μm to construct the 3D image, as shown in Figure 5a. A depth difference of 169.4 μm was observed statistically for the given imaging area, which is almost similar to the thickness value of the cover glass, as mentioned above. This result of a clear 3D image implies that an accurate measurement of transparent material can be successfully achieved even at a high-speed of 5 ms single axial scanning per pixel. Thus, the corresponding experimental results prove that our system shows a stable response in the mechanically lateral scanning process over the dimensions of 2.6 mm × 1.0 mm while maintaining a fast and accurate optical axial scanning capability of approximately 0.2 mm.

Subsequently, as shown in Figure 5b, the 3D surface profile of a “US cent” coin was measured using the same all-fiber SS-CCM configuration. Bidirectional scans along an area of 8.0 mm × 2.0 mm corresponded to 80 px × 20 px, respectively, with 5 ms of single axial scanning. The collected 3D image data corresponding to each depth were filtered using noise rejection algorithms to clearly isolate the finely protruding “cent” characteristics from the background. This clear imaging result confirms that single high-speed detection at 5 ms per pixel was achieved accurately, even for complex, fine features.

## 6. Conclusions

In this paper, a novel SS-CCM configuration incorporating a wavelength-swept laser was presented, and its utility for 3D surface profiling was explored. By adopting the functionality of the focal distance shift, similar to that used in the conventional CCM, the proposed system was demonstrated to exhibit chromatic dispersion linearity using the designed chromatic probe to achieve a highly efficient source-based spectral encoding. Compared to the conventional CCM, our SS-CCM system shows a highly stable behavior over a large range of acquisition time down to 5 ms. By obtaining 3D images using the SS-CCM system, axial measurements of target surfaces with a high-speed of 5 ms per pixel can be reliably extended to bidirectional lateral scanning while achieving an excellent lateral resolution. These results prove that the proposed SS-CCM can be employed as a novel strategy for overcoming the tradeoff between measurement speed and accuracy, which is a limitation of the existing CCM technology. We expect that this technology can be extended to the other spectral regions such as the O-band around 1310 nm and the C-band around 1550 nm because the swept-source and PD in those regions are already available commercially. In particular, the proposed concept of the replaced spectrometer by swept-source will be even more valuable in those longer-wavelength regions because the InGaAs-based-spectrometers for the O- and C- bands are much more expensive with a much smaller number of pixels, compared to Si-based spectrometry for the 800 nm band.
